# Effects of Anti-Angiogenesis on Glioblastoma Growth and Migration: Model to Clinical Predictions

**DOI:** 10.1371/journal.pone.0115018

**Published:** 2014-12-15

**Authors:** Elizabeth Scribner, Olivier Saut, Paula Province, Asim Bag, Thierry Colin, Hassan M. Fathallah-Shaykh

**Affiliations:** 1 Department of Mathematics, The University of Alabama at Birmingham, Birmingham, Alabama, United States of America; 2 Department of Mathematics, University of Bordeaux, Talence, France; 3 Department of Neurology, The University of Alabama at Birmingham, Birmingham, Alabama, United States of America; 4 Department of Radiology, The University of Alabama at Birmingham, Birmingham, Alabama, United States of America; University of Michigan School of Medicine, United States of America

## Abstract

Glioblastoma multiforme (GBM) causes significant neurological morbidity and short survival times. Brain invasion by GBM is associated with poor prognosis. Recent clinical trials of bevacizumab in newly-diagnosed GBM found no beneficial effects on overall survival times; however, the baseline health-related quality of life and performance status were maintained longer in the bevacizumab group and the glucocorticoid requirement was lower. Here, we construct a clinical-scale model of GBM whose predictions uncover a new pattern of recurrence in 11/70 bevacizumab-treated patients. The findings support an exception to the Folkman hypothesis: GBM grows in the absence of angiogenesis by a cycle of proliferation and brain invasion that expands necrosis. Furthermore, necrosis is positively correlated with brain invasion in 26 newly-diagnosed GBM. The unintuitive results explain the unusual clinical effects of bevacizumab and suggest new hypotheses on the dynamic clinical effects of migration by active transport, a mechanism of hypoxia-driven brain invasion.

## Introduction

In 1971, Folkman proposed that the growth of tumors depends on angiogenesis [1], [2]. This hypothesis catalyzed the development of anti-angiogenic therapy; several angiogenic targets have been identified including vascular endothelial growth factor (VEGF) [3], [4]. The first successful use of anti-angiogenic (AA) therapy was in a case of pulmonary hemangiomatosis [5]. Glioblastoma multiforme (GBM) is a malignant brain tumor that exhibits a classical multi-layer structure, which consists of a necrotic core (*ie* an area with no living cells), a rim of proliferative cells, and a margin of invasive cells [6], [7]. Two recent, large phase III clinical trials randomized newly-diagnosed GBM patients to standard of care with or without bevacizumab, a humanized anti-VEGF monoclonal antibody [8]–[10]. Unfortunately, this AA therapy did not prolong overall survival times (OS). However, both trials showed that patients treated with bevacizumab experienced a prolongation of progression-free survival times (PFS) and better quality of life. The prolongation of PFS reached statistical significance in the Roche trial [8], [9]. To better understand these unusual clinical results, it is fundamental that we gain a better insight into how GBM reacts to anti-angiogenic therapy.

GBM typically appears on MRI as a region of contrast-enhancing (CE) mass enclosing a necrotic area. The CE mass is surrounded by a diffuse nonenhancing (NE) region of abnormal T2/FLAIR signal. Kelly *et al.* obtained biopsies from the NE regions of the brain of newly diagnosed GBM patients; the pathological examination revealed tumor cells within intact parenchyma [11]. Recently, Gill *et al.* examined biopsy samples obtained from NE regions of GBM patients, which showed histological features of diffusely infiltrating gioma with neoplastic cells intermingled with nonneoplastic cells [12]. Furthermore, data from RNA-seq revealed that the NE regions are enriched in genes derived from the infiltrating tumor cells. Hammoud *et al.* reported that the amount of tumor necrosis on preoperative MRIs of 48 GBM patients was a predictor of short survival times [13]. Zhang *et al.*, Schoenegger *et al.*, and Pope *et al.* reported that large peritumoral edema in GBM is associated with poor outcome [14]–[16]; notably, a large ratio of NE to tumor volumes is an independent unfavorable prognostic factor [14]. Zinn *et al.* stratified GBM tumors by FLAIR volumes and analyzed the low and high FLAIR groups by gene and microRNA expression profiling and found that GBMs in the high FLAIR group are enriched in genes and microRNAs involved in cellular migration and invasion [17]. The mechanism of brain invasion by GBM cells is a fundamental question.

Keunen *et al.* studied GBM xenografts in animal brains and showed that treatment with bevacizumab lowered blood supply but was associated with an increase in infiltrating tumor cells [18]. Tang *et al.* reported that 4/8 glioblastoma cell lines exhibit a phenotype of low-oxygen-induced accelerated brain invasion mediated by activation of c-src and neural Wiskott-Aldrich syndrome protein [19]. Interestingly, the threshold of oxygen that controls the phenotypic switch is higher than what is typically anticipated for cancer-related hypoxia (*ie* 0.3%–1%); in fact, the enhancement in motility is observed at 5% as well as 1% ambient oxygen. Plasswilm *et al.* showed that hypoxia significantly increases motility of a glioblastoma cell line in an *in vivo* chicken model [20]. Furthermore, bevacizumab appears to enhance motility through its actions on VEGF, as the latter negatively regulates GBM cell invasion and motility through suppression of HGF-dependent MET phosphorylation [21]. It is important to note the recently-published results of Baker *et al.*, which demonstrate that, in the absence of angiogenesis, GBM cells migrate towards existing normal microvessels and grow in the perivascular spaces [22].

To decode the reaction of GBM to AA therapy, we previously constructed a mathematical model of GBM growth and hypoxia-driven brain invasion and simulations supported an exception to the Folkman Hypothesis [23]. To better understand how brain invasion affects key radiological features of GBM, we then constructed a concise model at the scale of clinical MRI, which includes a small number of equations and embodies two different mechanisms of brain invasion. The presentation is organized as follows; we start by detailing the assumptions and then proceed to a non-technical illustrative description of the mathematical model.

We show that simulations replicate key radiological features of GBM. Unexpectedly, the model suggested a new pattern of tumor growth in the absence of angiogenesis. This prediction was found to be true in 11/70 bevacizumab-treated patients whose images show expansion of the necrotic and FLAIR areas either in the absence of or without new Gadolinium enhancement. Interestingly, bevacizumab-treated tumors, without this pattern of expanding necrosis and FLAIR, are associated with better prognosis. The apparent mechanism for this tumor enlargement without angiogenesis is a cycle of growth and brain invasion that expands necrosis by depleting existing oxygen/nutrients. Simulations also predicted a positive correlation between the areas of FLAIR signal and necrosis; we looked for that correlation in retrospective clinical experiments and found it to be true in 26 patients with untreated GBM. Simulations and clinical results are presented side-by-side.

The clinical results enhance our confidence in the model, which offers a possible mechanistic explanation of the effects of bevacizumab on improving patient-reported outcomes and PFS without prolongation of OS: though AA therapy reduces the tumor mass and slows progression, it expands brain invasion leading to rapid recurrence when the tumor acquires resistance. The model also suggests a new hypothesis that links the mechanism of tumor cell migration to clinical features: GBM tumors that react to hypoxia by migration towards existing blood vessels in normal brain are associated with large areas of FLAIR signal both before and after treatment with bevacizumab.

## Materials and Methods

### Description of the Model Equations

The system of equations is
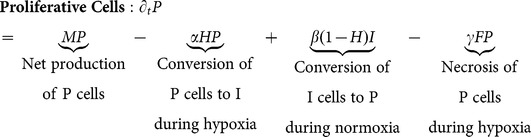
(1)


**Invasive Cells:**

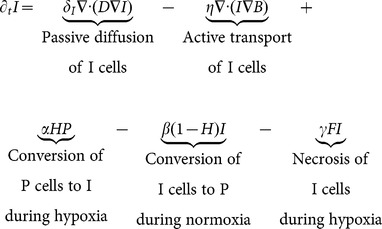
(2)

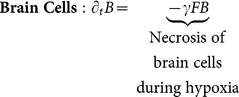
(3)

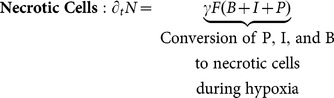
(4)


Assumptions regarding angiogenesis, hypoxia, mitosis, and necrosis:
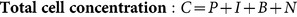
(5)

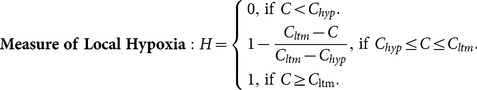
(6)

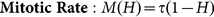
(7)

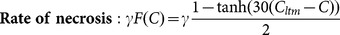
(8)

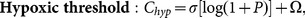
(9)

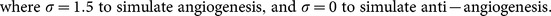


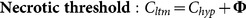
(10)


**Figure 1:**
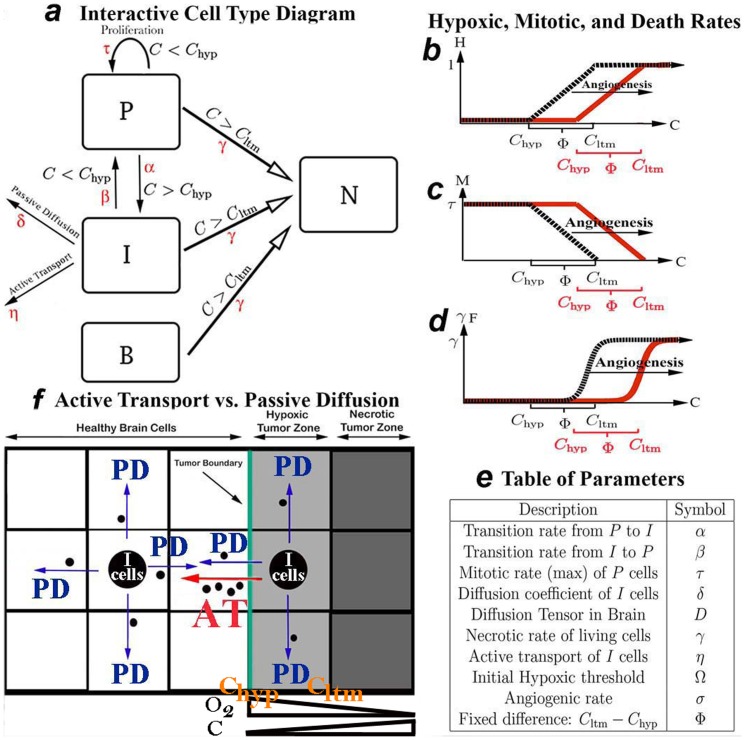
Illustrative cartoons and description of the parameters. (a) Interactive Cell Type Diagram with Parameters. Parameters driving different types of cell movements or transitions are shown in red.(b) Hypoxia (H), (c) Mitotic Coefficient (*M*), and (d) Necrotic Rate (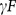) as a function of 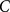 and angiogenesis. (e) Description of Model Parameters. (f) Active Transport (AT) verses Passive Diffusion (PD). AT (red arrow) actively drives I cells in bulk towards normal healthy brain. AT does not occur once the cells reach a new region of healthy brain cells as the concentration of these cells is now uniform (*i.e.* the gradient of B is zero). PD (blue arrows) allows the I cells to diffuse down their concentration gradient equally in all directions other than the necrotic zone. The model assumes that the local oxygen concentration is inversely related to 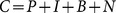; furthermore, 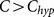 causes the phenotypic switch 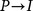 while 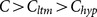 activates rapid necrosis.

A description of the parameters may be found in Fig. 1.

### Patient Data

The clinical research was approved by the Institutional Review Board (IRB) of the University of Alabama at Birmingham. The IRB waived the requirement of obtaining a signed informed consent document because the research presents no more than minimal risk of harm to participants and involves no procedures for which written consent is normally required outside of the research context. Patient information was anonymized and de-identified prior to analysis.

#### GBM tumors at first recurrence: expanding necrosis

We reviewed the records of a total of 69 patients diagnosed with GBM and 1 patient with gliosarcoma, treated at the University of Alabama at Birmingham by bevacizumab (10 mg/kg every 2 weeks) at first recurrence between 2008 and 2013. Only 23 records included all the following sequential MRIs (see S1 Figure),

1. MRI documenting first recurrence in the absence of bevacizumab,
2. MRI showing maximal effects of bevacizumab causing reduction in Gadolinium enhancement and FLAIR signal abnormality,
3. MRI showing a tumor without new or increased enhancement as compared to the previous MRI (see (2) above),
4. MRI documenting the development of new enhancement on bevacizumab.

### Numerical methods

Simulations were obtained using finite difference schemes. The brain was discretized into a 127×127 spatial mesh with each cell measuring approximately 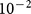 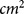 in area, and a small concentration of both proliferative cells (on the order of 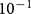) and invasive cells (on the order of 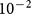) was inserted into a single cell at the start of the simulation. The initial topography of the brain was taken from a virtual MRI slice and included brain matter as well as skin and bones, which are assumed impermeable to the migrating tumor

(http://brainweb.bic.mni.mcgill.ca/brainweb/).

### Statistical analysis

The Kaplan-Meier survival analysis was done using JMP (www.jmp.com). The *R*-square was computed using Matlab (www.mathworks.com).

## Results

### Assumptions and model

Several cellular types are considered (see interactive diagram in Fig. 1a). Proliferative cells, denoted by 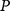, are glioma cells that are actively dividing and do not move. Invasive cells, 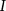, migrate but do not divide. Brain matter cells, 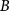, begin at an initial fixed concentration; they do not divide or migrate. If the hypoxia is severe, cancer cells 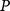 and 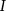, as well as brain cells 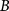, eventually die; this is called necrosis 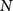. The total number of cells, C, is taken as the sum of 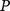, 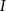, 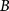, and 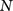 (Equation 5).

Our main assumption is that GBM cells switch from one phenotype to another (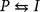), depending on the local hypoxic state in the tissue. Hypoxia causes 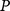 cells to stop dividing and switch to 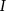 cells, which have the ability to leave the core of the tumor and invade the brain. Due to their important mobility, 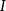 cells flee the hypoxic area; after traveling in the brain and reaching a favorable local environment, 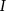 cells stop their movement and become proliferative again, leading to tumor growth. These assumptions are called the “go-or-grow” phenotype [24]. Therefore, one has to model the local quantity of nutrients, the ability of cells to divide, move, or die, and the tumor's ability to form new blood vessels and hence capture more nutrients and oxygen.

Modeling oxygen diffusion, new blood vessel formation, chemotaxis, and other processes associated with angiogenesis is not only computationally consuming, but also unrealistic at the scale we are considering (*i.e.* medical images). We instead allow the available oxygen/nutrient supply to vary indirectly with the total concentration of cells 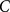 (Equations 5–6); furthermore, we assume that angiogenesis elevates a key local hypoxia threshold, which varies directly with 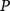 (Equation 9).

For simplicity, we define the local hypoxic state 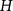 (Equation 6) in any specific brain location as a function of 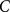, and we identify 2 main critical thresholds: 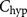 (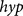 for hypoxia), when cells begin slowing their growth rate and switching from one phenotype to another, and 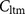 (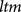 for lethum), when cells begin to die (Figs. 1b–1d). The mitotic rate 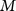 varies spatially depending on 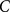 (*i.e.* hypoxia, see Figs. 1b–1d and Equation 7). 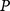 cells divide at their maximal rate when 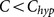. The mitotic rate decreases and is inversely proportional to the local hypoxic state when 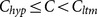; and it eventually vanishes when 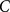 exceeds 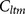, which triggers necrosis (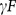, see Fig. 1d and Equation 8). Hence, the measure of local hypoxia in the brain is key to controlling (1) the conversion of the tumor from one phenotype to another (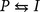), (2) tumor cell movement, as invasive cells tend to seek areas with more nutrients and a higher oxygen supply (see below), and (3) the growth and death rates of tumor and brain cells. The density 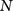 is computed by collecting dead cells of all types (P, I, and B; see Equation 4). For the purpose of simulation, regions of the brain are considered dead when 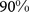 or more of the initial brain cells have converted to 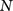 cells.

Angiogenesis elevates the thresholds 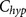 and 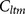 as a function of 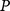 (Equations 9–10 and Figs. 1b–1d), thus supporting a denser tumor before the death rate reaches its maximum 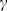. Anti-angiogenic treatment increases local hypoxia in the tumor by limiting the supply of oxygen and nutrients. In our model, we emulate this treatment by fixing and thus preventing the increase of the thresholds 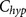 and 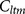 (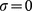, see Equation 9). In this way, AA treatment acts to lower these 2 thresholds in an angiogenic tumor, which sooner suppresses the mitotic rate of P cells and hastens necrosis and the conversion of P to I cells.

The concentration of 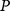 cells is modeled by Equation 1, which corresponds to our assumptions (Fig. 1a): 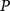 cells divide, switch to an invasive phenotype (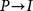) or die as a function of the level of local hypoxia (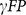). Furthermore, they appear and subsequently divide when invasive cells switch back to the proliferative phenotype (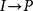), *ie* when 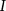 cells reach new regions of low cellular density (and hence higher levels of nutrients). Note that P cells switch to I cells at a rate 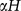 whose domain is 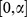. Hence, when the hypoxic state is low, *ie* 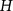 is close to 0, there is no or little switching of proliferative cells to the invasive phenotype. When the hypoxic state is high, *ie* 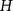 is close to 1, the transition of 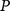 to 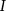 (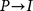) occurs at the maximum rate 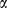. Conversely, 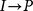 occurs at a rate 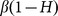, which is elevated when hypoxia is low, 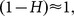 and depressed when hypoxia is high, 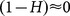.


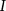
 cells alone are responsible for the tumor's ability to invade the brain; in our model they follow a partial differential equation (in time and space), where there is no cellular division, and which includes 2 terms for migration (Equation 2). 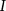 cells are only produced by proliferative cells under hypoxic conditions. The first migration term, 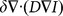, describes passive diffusion (PD), defined by Fick's Law, which states that the rate of movement of the invasive cells is proportional to its own concentration gradient (see illustration in Fig. 1f), *i.e.* away from areas of higher 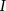 concentrations to areas of lower 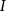 concentrations at a rate 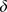 irrespective of nutrient availability. Another parameter, 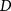, varies spatially to replicate the increased rate of movement of cancer cells along white matter tracks in the brain. Ultimately, the values for 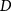 will depend on the individual topography of the brain, allowing for more patient-specific tumor simulations. Combined, this passive diffusion term translates into increased tumoral movement along white matter tracks in all directions away from the areas of highest invasive cell concentrations, which usually occurs on the boundary of tumor, hence contributing to further tumor expansion. White matter tracks (bundles of axons) are assumed dead (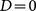) at the necrotic core of the tumor (defined by 90% or more brain death), and hence invasive cells will not diffuse back into the dead center of the tumor.

The second migration term, 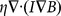, reflects the preferential movement of cells in the direction towards areas with the highest number of healthy brain cells (Active Transport, AT); the speed of migration is proportional to the concentration of 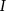 cells and the gradient of B, 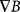 (see Fig. 1f). For example, for invasive cells located on the hypoxic edge of the tumor where the concentration of brain cells in one direction is steeply increasing (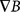), 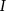 cells move away from the tumor core by AT in search of more nutrients. Likewise, as invasive cells approach healthy regions of the brain where the concentration gradient of 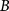 cells is close to 0, speed of movement in that direction will decrease. In this way, the gradient of B is a measure of the local hypoxia and death in the brain. Also, because AT models bulk movement of a biological species, higher concentrations of invasive cells lead to greater mass transport in regions of increasing brain density, such as the boundary of a hypoxic region in the brain. In this way, both the gradient of B and the density of I determine the bulk movement of I cells during AT. Fig. 1f illustrates the essential differences between AT and PD of the invasive cell types.

In further, we assessed the effects of AT alone or PD alone by setting 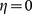 or 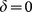 (Equation 2, respectively. As shown below, each term alone will allow our model to behave accurately. Nevertheless, AT provides key characteristics of the tumor.

### Model replicates multi-layered structure of GBM

**Figure 2:**
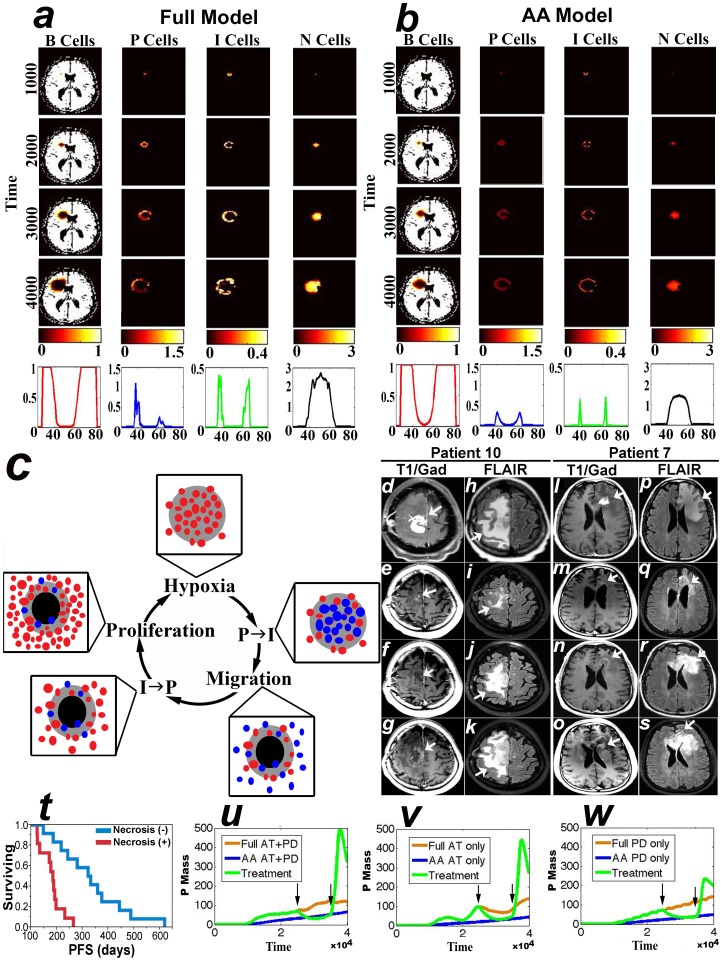
GBM growth in the presence and absence of angiogenesis. (a) Multilayer Structure of GBM in the presence of angiogenesis (Full model). (b) Multilayer structure of GBM of the AA model. The last rows of (a) and (b) plot a vertical slice, along the y-axis of the corresponding 4 cell-type concentrations at the final time step. (c) cartoon depicting the cycle of tumor growth and brain invasion that expands necrosis in the absence of angiogenesis. Gray and black areas represent hypoxia and necrosis, respectively. Red balls are 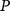 cells and blue balls are 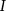 cells. (d)–(s) are MRIs of 2 GBMs with expanding necrosis on bevacizumab; (d)–(g) and (l)–(o) are Gadolinium-enhanced T1-weighted images (T1/Gad); (h)–(k) and (p)–(s) are FLAIR images (arrows). The first row demonstrates first recurrence before bevacizumab (arrows). The second row shows the maximal effects of bevacizumab. The third row shows expanding necrosis without significant new enhancement (arrows in f and n) but with enlargement of the FLAIR signal (arrows in j and r). The fourth row shows tumor progression on bevacizumab. Clinical details related to (d)–(s) can be found in S1 Text. (t) is a Kaplan-Meier analysis of the progression free survival times (PFS) of patients with (Necrosis +, 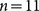) and without (Necrosis -, 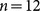) expanding areas of necrosis (Log-Rank 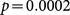). (u)-(w) show the results of the simulations of the effects of, respectively, AT + PD, AT, and PD on the Full (orange), AA (blue, *ie* 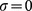 starting from time 0), and treatment (green) models. The latter consists of the Full model until time step  = 2500 (first black arrow) when AA is applied and then lifted at time step  = 3500 (second black arrow). Time units are arbitrary.

The Full model includes angiogenesis and simulates untreated GBM; in Fig. 2a, the Full model is configured to include AT + PD; simulations reproduce the multi-layered structure as shown in Fig. 2a, which represents a two-dimensional slice of a GBM, as might be observed in a patient's MRI. The last line displays a one-dimensional vertical cross-section of the different cell concentrations at the end of the simulation. The tumor is initially a spheroid composed entirely of 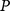 cells. After a while, the cell population reaches the threshold 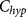 and some 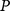 cells are switching to the invasive phenotype as seen in the second column of Fig. 2a. Then, inside the core of the tumor, the total population reaches 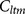, and cells start dying: a necrotic core appears at the center of the proliferative rim, as can be seen in the first and fourth columns of Fig. 2a. Capturing the heterogeneity of tumor and brain cell types is important in understanding the roles of passive diffusion and active transport, as well as for replicating various dynamics and behaviors observed in GBMs.

### GBM grows without angiogenesis by expanding necrosis: simulations and clinical validation

The AA model simulates the growth of GBM in the absence of angiogenesis, *ie* following the use of bevacizumab; in Fig. 1b the AA model is configured to include AT + PT. Observe that the size of the tumor is smaller and that the concentration of P cells in the proliferative ring is less than that in the Full model due to the inability of the tumor to secure new sources of oxygen/nutrients. The peak concentrations of invasive cells and necrotic cells are also less than that of the Full model. Also note that in the AA model the death of 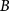 cells is considerably less than what is observed in the Full model. Nevertheless, the results of the simulations also predict that a GBM tumor may continue to grow in the absence of angiogenesis (AA model), albeit at a slower pace than in the presence of angiogenesis (Full model with angiogenesis). Furthermore, this growth manifests by an expanding area of necrosis (see fourth column of Fig. 2b), which is driven by the cycle of growth and hypoxia-driven brain invasion depicted in Fig. 2c. Rapid proliferation and division of 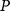 cells generates hypoxia and eventually necrosis by depleting local nutrients/oxygen, thus causing the 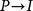 switch and migration of 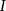 cells towards healthy brain where the 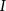 cells revert back to 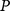 (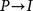).

We validated the results of the simulations of Fig. 2b by testing the hypothesis that GBM continues to grow in the human brain during anti-angiogenic therapy. We reviewed the MRIs of 69 patients, diagnosed with GBM and one patient with gliosarcoma, treated with bevacizumab at first recurrence. Because bevacizumab typically causes an initial decrease in both Gadolinium enhancement and FLAIR signal changes, we looked for patients whose imaging shows at least one stable MRI after the maximal effects of bevacizumab are observed. Only 23/70 patients met this criterion (see Table 1 and S1 Figure). As predicted by the model, subsequent images of 11/23 patients demonstrated expanding areas of both necrosis and FLAIR in the absence of or without new significant Gadolinium enhancement (Necrosis (+), Figs. 2d–2s). Note that the increase in peri-tumoral FLAIR in Figs. 2h–2k and 2p–2s are also consistent with the results of the simulation of the AA model, which show an increase in 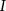 cells with time (Fig. 2b). Later imaging of these 11 Necrosis (+) patients confirmed tumor recurrence by development of new Gadolinium-enhancement at the same site of expanding necrosis and FLAIR (Figs. 2g and 2o); the average time interval between the development of expanding necrosis and new enhancement at the same site was 60 days (std  = 21 days).

**Table 1:** Characteristics of patients with GBM at first recurrence.

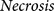	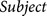	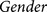	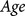	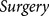	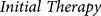	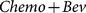
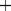	1	M	27	R	XRT/Tem	CCNU
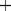	2	M	56	R	XRT/Tem	None
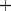	3	M	37	Biopsy	XRT/Tem	None
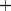	4	M	53	GTR	XRT, Tem	Tem
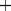	5	M	43	Biopsy	XRT/Tem, ICT-107	None
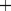	6	M	47	GTR	Gliadel, XRT/Tem	CCNU
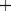	7	M	55	GTR	XRT/Tem, R-(-)-gossypol	CPT-11
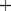	8^**^	M	53	GTR	XRT/Tem	None
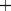	9	F	50	R	XRT, Tem	Tem
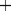	10	F	44	Biopsy	XRT/Tem	CCNU
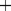	11	F	73	STR	XRT/Tem	CCNU
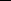	12	M	57	STR	XRT/Tem	CPT-11
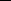	13	M	65	GTR	XRT/Tem	CPT-11
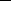	14	M	29	STR	XRT/Tem	CCNU + Etoposide
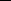	15	M	54	STR	XRT, Tem	CCNU
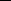	16	M	61	STR	XRT/Tem	Tem
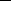	17	M	53	STR	XRT/Tem	CPT-11
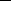	18	M	63	STR	XRT/Tem	None
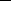	19	M	53	GTR	XRT/Tem	Tem
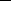	20	M	38	GTR	XRT/Tem	CPT-11
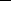	21	M	65	STR	Gliadel, XRT/Tem	Tem
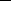	22	F	58	R	XRT/Tem	None
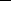	23	F	60	STR	XRT/Tem	None

Necrosis (+) and (-) indicate that the tumor showed, respectively, the presence or absence of expanding area of necrosis (see Fig. 2). M =  Male. F =  Female. Age  =  age at initial diagnosis in years. Initial Therapy  =  therapy given prior to first recurrence. Surgery  =  initial surgical procedure at the time of diagnosis. Chemo + Bev  =  chemotherapy given with bevacizumab. XRT  =  radiation therapy. Tem  =  Temozolomide. XRT/Tem  =  radiation therapy with concurrent Temozolomide. XRT, Tem  =  radiation therapy followed by Temozolomide. CCNU  =  Lomustine. CPT-11 =  Irinotecan. STR  =  subtotal resection. ICT-107 =  ICT-107 vaccine. Gliadel  =  Gliadel wafers. GTR  =  gross total resection. R =  craniotomy surgical details unknown. ** =  This patient had a diagnosis of gliosarcoma. The mean age of patients 1–11 is 48.4 years. The mean age of patients 12–23 is 54.7 years.

The patient data, which are consistent with model predictions (see Fig. 2b), support the conclusion that GBM grows during anti-angiogenesis by expanding necrosis and FLAIR. Furthermore, this pattern of expanding necrosis and FLAIR predicts a poor prognosis; note that the 11 Necrosis (+) patients have a significantly shorter PFS, as compared to the 12 patients without this pattern of recurrence (Necrosis (-), Fig. 2t). The mean values of the PFS are 333 and 178 days for the Necrosis (-) and Necrosis (+) groups, respectively (Log-Rank 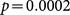).

### Reproducing the anti-angiogenic rebound effect

Typically, patients experience an initial decrease in enhancement and FLAIR after the start date of bevacizumab (see Figs. 2d–2s). Interestingly, some bevacizumab-treated GBM tumors exhibit a rapid rebound when the tumor acquires resistance to the drug or when bevacizumab is discontinued [23], [25]. Hence, we study a treatment model, which applies anti-angiogenic therapy in a growing tumor at time step 2500 (*ie* 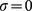) and then lifts it at time step 3500 (black arrows). Figs. 2u–2w simulate the effects of AT alone (*ie* 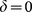), PD alone (*ie* 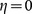), and AT + PD on the growth of the proliferative tumor mass, *P*, in the Full (orange), AA (blue), and treatment (green) models. The results reveal that either AT alone or PD alone generates a rebound rapid tumor growth when the tumor becomes resistant to AA therapy. Note also that the model replicates the immediate effects of bevacizumab on decreasing the Gadolinium-enhancing tumor (see Fig. 2e); bevacizumab treatment lowers the mass of 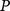 cells causing a shift from the growth curve of the tumor (green) from the Full model (orange) to the curve of the AA model (blue) (Figs. 2u–2w).

### Comparing active transport to passive diffusion: model simulations and clinical validation

**Figure 3:**
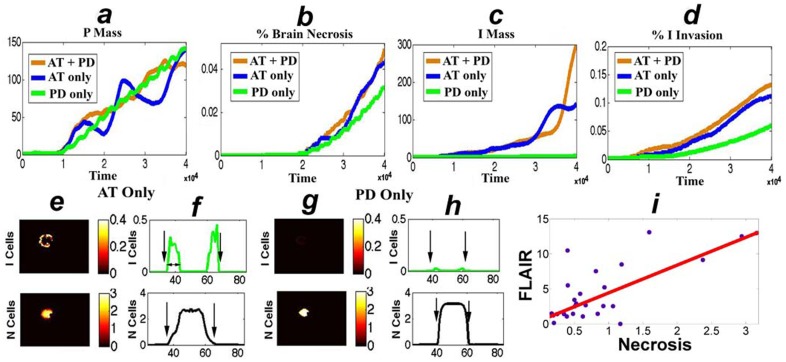
AT enhances both necrosis and invasion. The first row compares the effects of AT only (blue), PD only (green), and AT + PD (orange) on the evolution of the proliferative tumor mass (a, P Mass), percent of the brain that is necrotic (b), invasive tumor mass (c, I Mass), and percent brain invasion by I cells (d) for the Full model. (e) and (g) show the 2-dimensional distribution of 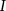 cells and 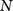 at the final steps of the AT only and PD only models, respectively; (f) and (h) plot vertical one-dimensional sections. (i) plots the areas of necrosis vs (FLAIR signal - Tumor) (*mm* ^2^, see S2 Figure) of 26 newly-diagnosed GBMs. The data is fitted to the polynomial 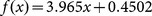 by the robust least square method (least absolute residuals, R-square: 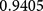). Units are arbitrary.

The simulations, shown in Figs. 2a–2b, include AT + PD; to better understand the contribution of AT and PD, we configured the Full model for AT only (*ie* 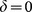), PD only (*ie* 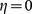), and AT + PD and examined the P tumor mass, I tumor mass, and the percentage of the brain that is necrotic (percent necrosis) or invaded by at least 10^−4^ *I* cells (percent invasion). Note that parameters 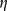 (AT) and 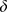 (PD) were chosen such that the proliferative tumor masses at the final time step were roughly equivalent for the AT-only and PD-only simulations (Fig. 3a). For the same mass of 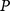 cells in the Full model, AT generates a higher mass of 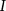 cells, and larger areas of brain invasion and necrosis than PD only (see Table 2 and Figs. 3b–3h).

**Table 2:** Summary of Tumor Response under AT only verses PD only in the Full and Treatments models.

	I Concentration	I Distribution in Space	Percent Necrosis
Full model	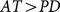	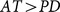	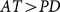
Treatment Model	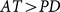	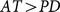	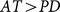

**Figure 4:**
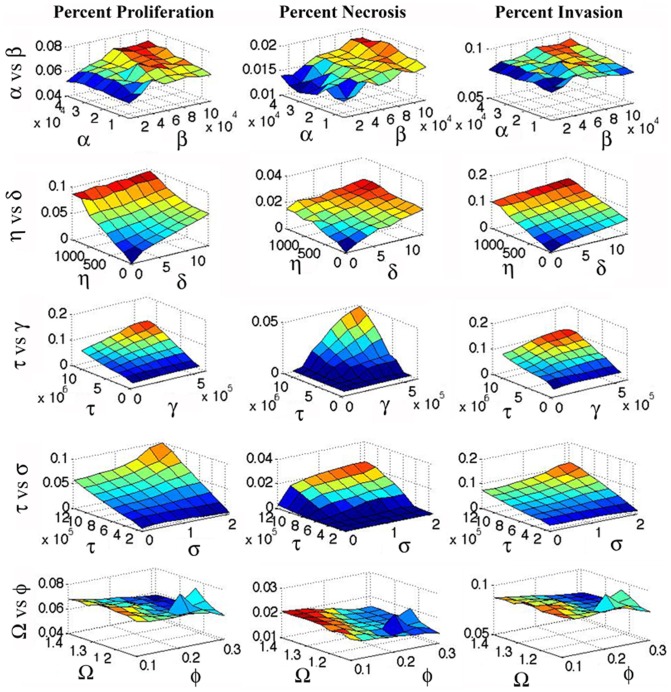
Sensitivity Analysis. Percent Proliferation (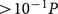), Percent Necrosis (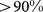 brain death), and Percent Invasion (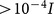) for different parameter choices at time  = 3000. 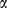, transition rate from 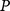 to *I*. 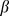, transition rate from 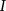 to 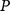. 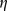, active transport of 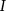 cells. 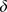, diffusion coefficient of 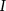 cells. 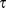, mitotic rate of 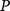 cells. 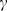, death rate of living cells. 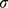, angiogenic rate. 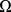, initial hypoxic threshold. 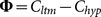.

Figs. 3b and 3d predict a positive association between the size of the areas of necrosis and brain invasion by 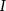 cells in both the PD-only (green curves) and AT-only models (blue curves). To further investigate this correlation, we performed a parameter sensitivity analysis by varying parameter pairs and measuring Percent Necrosis and Percent Invasion; the findings, shown in Fig. 4, also support a positive association between the areas of necrosis and brain invasion by 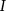 cells. To validate this association, we examined the MRIs of 26 untreated GBM (see S2 Figure).

The rationale for choosing untreated GBM is as follows. Pathological evaluations of brain biopsies of untreated GBM identified tumor cells in the peritumoral NE regions [11], [12]. In addition, radiation therapy protocols that treat the high FLAIR signal are associated with improved outcomes [26]. The aforementioned observations support the assumption that, in untreated GBM, the area/volume of brain, consisting of FLAIR/T2 signal changes, is invaded by tumor cells. The MRIs of the 26 patients with newly-diagnosed untreated GBM were obtained prior to any surgical procedures; they were selected if the 2-dimensional axial slice is the direction that includes the maximal FLAIR-signal (see S2 Figure). Fig. 3i plots the area of (FLAIR signal - Tumor) vs. area of necrosis; the results support the hypothesis that large necrotic areas in untreated GBM, correlate positively with large areas of high FLAIR signal (R-square 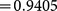). These findings are consistent with the model prediction.

As compared to PD, AT enhances both necrosis and brain invasion in untreated GBM (see Table 2 and Figs. 3b-3h). Next, we apply the treatment model to evaluate the potential roles of AT and PD in the pathogenesis of the new pattern of progression by expanding necrosis and FLAIR (Fig. 2). The findings reveal that AT alone replicates all the features of the progression, as it elevates I cell mass, and expands necrosis and brain invasion by I cells (see Table 2 and S3b Figure). PD alone causes minimal effects on the mass of I cells (S3c Figure). These results support the hypothesis that AT alone plays a key role in generating the pattern of progression described in the 11 patients with Necrosis (+) (see Table 2 and Fig. 2).

## Discussion

The cycle of proliferation and hypoxia-driven brain invasion defines an exception to the Folkman hypothesis that underlines the importance of a better understanding of the molecular mechanisms of invasion and AT, as well as the need to develop biomarkers for AT and anti-motility drugs. Nowosielski *et al.* define 4 patterns of recurrence of primary GBM treated by bevacizumab and report differences in survival times [27]. Our results suggest a new pattern of recurrence/resistance to bevacizumab, associated with poor survival times, that manifests by an expanding areas of necrosis and FLAIR in the absence of significant enhancement (Figs. 2c–2t).

Some GBM tumors exhibit enhanced motility at 5% ambient oxygen, which is higher than the typical 0.3–1% concentrations observed in cancer hypoxia (see [28]). Hence, it is reasonable to assume that GBM cells sense and react to low levels of oxygen that are higher than the severe necrosis-causing hypoxia in the center of the tumor. We assume hypoxia to be positively related to cellular density such that it causes a switch from 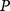 to 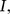 when it exceeds the threshold 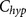 and it causes rapid death when it surpasses 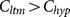. Notice that hypoxia initiates the phenotypic change (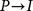) before it reaches severe levels that cause necrosis, *ie.* when 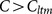. The model incorporates the idea of the go-or-grow phenotype; that is, 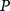 cells replicate but do not move and 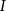 cells migrate but do not divide. When the cellular densities exceed 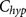, 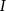 cells are formed and they start their migration by 2 mechanisms: 1) preferentially towards the direction of normal brain, which includes existing blood vessels (AT), and 2) equally in all directions other than towards necrosis (PD). 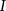 cells start switching back to the proliferating phenotype when the local cellular density is below 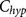, *ie* effectively when the 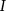 cells cross the boundary of the tumor into brain tissue. Our model is consistent with the results of Baker *et al.* whose results support the idea that GBM cells preferentially migrate towards existing, normal microvessels and that they grow in the perivascular spaces [22]. Note also, that hypoxia contributes not only to peripheral brain invasion but also to peripheral neo-angiogenesis when 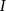 cells switch back to 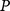 cells, which are the drivers of angiogenesis. Recent molecular evidence supports the existence of the 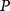 and 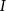 cells proposed by the go-or-grow phenotypes; Tan *et al.* found that microRNA-9 inhibits proliferation but promotes migration, whereas cyclic AMP response element-binding protein (CREB) exerts a pro-proliferative and anti-migratory effect [29]. In addition, Höring *et al.* found that carboxypeptidase E levels in GBM cells were associated with high proliferative and low migratory rates [30].

Our model assumes that normal brain cells, 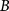, do not divide or migrate; nonetheless, normal astrocytes and microglia may divide, migrate, and invade the tumor, thus these cells could potentially contribute to tumor hypoxia. Notice, though, that such a contribution to tumor hypoxia is incorporated in the model by setting the key thresholds, 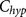 and 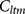. For example, tumor with a high density of microglia could be modeled by a lower value of the 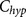 parameter.

Growing GBM tumors produce VEGF to promote angiogenesis, which provides a conduit for blood flow to deliver nutrients and oxygen in order to meet the metabolic demands of the growing neoplasm. In turn, as the tumor enlarges, necrotic and hypoxic zones are created in tumors due to the immature nature of tumor vasculature, especially if the speed of tumor replication exceeds the rate of angiogenesis [31]. Therefore, in certain regions of tumors, the vessels may be highly permeable and ectatic. These immature vessels do not provide nutritive flow thus contributing to the hypoxic environment, which reduces the sensitivity of tumor cells to radiotherapy and impedes the delivery of chemotherapeutic drugs. The Jain vascular normalization hypothesis stipulates that anti-angiogenic therapy could induce a *transient* normalization of the structure and function of some blood vessels in the tumor by improving vascular permeability, organization, and perfusion [32]. During this normalization window, which lasts from hours to days after VEGF blockade, there is improvement in tumor oxygenation, drug delivery, and radiation sensitivity. In the case of GBM, it appears that tumor cell-derived angiopoietin-1 is an absolute requirement for normalization [33]. Notice that the new pattern of recurrence, consisting of progression by necrosis and FLAIR (see Figs. 2u–2w), occurred much later than the transient normalization window because the selection of 23/70 patients required at least 1 stable MRI after the maximal effects of bevacizumab were observed (see S1 Figure). In fact, the median time interval between the start of bevacizumab and the development of the expanding necrosis and FLAIR signal changes was 128.5 days (minimum  = 77, maximum  = 202); therefore, it is reasonable to assume that, in these cases, bevacizumab augments hypoxia by causing a reduction in vessel density [31], [32]. Nonetheless, the question arises of the effects of the transient initial normalization period on necrosis and FLAIR [32]. To address this question, we model vascular normalization by increasing the value of the angiogenesis parameter, 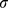, for a short period of time after the initiation of AA; the results reveal that both necrosis and 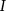 cell density increase as compared to simulations that apply AA without normalization (see S5 Figure).

VEGF is a potent mediator of vascular permeability and blood brain barrier disruption in brain tumors [34], [35]; thus, bevacizumab has been postulated to exert steroid-like actions in GBM [36]. Nonetheless, our simulations suggest that bevacizumab reduces the size of the contrast enhancing lesion at least in part by decreasing the number of proliferative cells (Figs. 2u–2w). Notice that, at the time of maximal effects of bevacizumab, the size of the area with FLAIR signal changes is smaller than the area of contrast-enhancement prior to initiation of therapy (see Figs. 2i vs. 2d, and Figs. 2q vs. 2l); this observation supports the idea that bevacizumab caused a reduction in tumor mass. This explains the improvement in patient-reported outcomes measured by the phase III clinical trials [8], [9]. The combination of growth along the AA curve, combined with the rebound growth when the tumor acquires resistance, supports the criticisms of the use of PFS as a diagnostic end-point in GBM (see Fig. 2 and [37]).

Furthermore, the rapid rebound in tumor growth/enhancement when the tumor becomes resistant to bevacizumab (Figs. 2u–2w) is an apparent justification of the lack of benefits on OS. This explosive growth in P cells (*ie* tumor enhancement) in some tumors appears to be mediated by 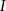 cells, whose concentrations and spatial distributions are augmented by the anti-angiogenic therapy (see Figs. 2–3, S3 and S4 Figure). When the tumor acquires resistance to bevacizumab, revascularization is re-initiated causing an increase in the local oxygen concentration and the phenotypic switch 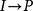, which leads to an explosive growth. Bergers and Hanahan have suggested different mechanisms of resistance to anti-angiogenic therapy leading to revascularization; these include: 1) evasive resistance to VEGF inhibitors caused by upregulation of alternate pro-angiogenic signals, and 2) intrinsic resistance due to non-responsiveness of a tumor to bevacizumab [38]. Note that the criteria for selecting the 23/70 patients (see S1 Figure) exclude tumors with intrinsic resistance to bevacizumab in the sense that the GBMs showed an initial response leading to reduction in enhancement and FLAIR (Fig. 2). Furthermore, our results suggest a new pattern of *dynamic resistance* to bevacizumab, which differs from the evasive and intrinsic types, as it is characterized by an initial decrease in proliferating cells followed by the go-or-grow dynamics leading to the cycle of migration and growth depicted in Fig. 2c.

Other models attempt to capture the dynamics of GBM; most model invasion by passive diffusion [39]–[42]. Swanson *et al.* has introduced the proliferation-invasion-hypoxia-necrosis-angiogenesis model (PIHNA) that stipulates the presence of 2 glioma cell populations in normoxic and hypoxic states [43]. A complex model, developed by Frieboes *et al.* [44], includes mechanical stress, interactions with extra-cellular matrix, angiogenesis, and growth-promoting factors. Neither the models of Frieboes or Swanson consider the go-or-grow phenotype. The model assumes that normal brain cells, *B*, do not divide or migrate; nonetheless, normal astrocytes and microglia may divide, migrate, and invade the tumor, thus contributing to tumor hypoxia. Notice that such a contribution to tumor hypoxia is incorporated in the model by setting the key thresholds, 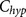 and 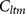. We have previously constructed a mathematical model of GBM growth and hypoxia-driven brain invasion that includes the go-or-grow phenotype and angiogenesis; simulations also support an exception to the Folkman hypothesis and generate key features of GBM including its multilayer structure and the rebound rapid growth [23]. The benefits of the current concise model include being amenable to analytical theory as well as producing significant savings in computational time such that parameter estimation from patient data is now a possibility.

Our findings reveal fundamental differences between AT and PD; in untreated tumors, AT enhances necrosis and brain invasion (Fig. 3) and produces higher concentrations of 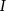 cells (Figs. 2–3, S3 and S4 Figure). Furthermore, the results shown in Table 2 and S3 Figure are consistent with the hypothesis that AT mediates the clinical pattern of progression by FLAIR and necrosis (see Fig. 2) as AT increases both the concentration of I cells and expands necrosis. This observation also raises the question whether AT is linked to the progression by FLAIR, as defined by the RANO (Response Assessment in Neuro-Oncology working group) criteria [45]. In summary, our results: 1) contribute a mechanistic explanation of the unusual clinical effects of bevacizumab, 2) identify a new pattern of progression/recurrence of GBM by expanding necrosis and FLAIR (Fig. 2 and 3) uncover a correlation between the size of necrosis and high FLAIR signal in untreated GBM (Fig. 3i). The clinical results in Figs. 2 and 3 set the stage for prospective trials that include a larger number of patients.

## Supporting Information

S1 Text **Legend of clinical imaging of** **Figs. 2d–2s** **.**
(PDF)

S1 Figure **Selection of the 23/70 patients.** Cartoon depicting the timing of the MRIs before and after first recurrence and the identification of 23/70 patients, treated by bevacizumab at first recurrence (see Table 1), who have a MRI with no new or increased enhancement after the MRI showing the maximal beneficial effects of bevacizumab on enhancement or FLAIR.
(TIF)

S2 Figure **Tumor, Necrosis and FLAIR Measurements.** An example of the measurements. (a) and (b) show the measurements of the largest diameters of the areas of enhancing tumor and necrosis, respectively. (c) shows the largest diameters of the areas showing FLAIR signal abnormality. The areas are computed by the product of the two perpendicular diameters. The measures plotted on the y-axis of Fig. 3i are FLAIR area - tumor area.
(TIF)

S3 Figure
**Comparison of the Treatment, Full, and AA Models.** The Full, AA (*ie* 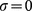 starting from time 0), and treatment curves are colored in orange, blue, and green, respectively. The latter consists of the Full model until time step  = 2500 (first black arrow) when AA is applied and then lifted at time step  = 3500 (second black arrow). Simulations of the AT + PD model are shown in (a), (d), and (g). Simulations of the AT only model are shown in (b), (e), and (h). Simulations of the PD only model are shown in (c), (f), and (i). The effects on I Cell mass, Percent Brain Invasion (*ie* brain including>10^−4^ I cells), and Percent Necrosis (*ie* areas including>90% necrosis) are shown in (a–c), (d–f), and (g–i), respectively. The effects on P cell Mass is shown in Figs. 2(u)–2(w). Time units are arbitrary.(TIF)

S4 Figure
**AT enhances brain invasion in the AA model.** The first row compares the effects of AT only (blue), PD only (green), and AT + PD (orange) on the evolution of the proliferative tumor mass (a, P Mass), invasive tumor mass (b, I Mass), and percent brain invasion by I cells (c) for the AA model. (d) and (e) show the 2-dimensional distribution of 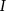 cells at the final steps of the AT only and PD only models, respectively. The parameters parameters 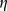 (AT) and 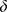 (PD) are the same as in Figs. 3a–3h. Units are arbitrary.(TIF)

S5 Figure
**Jain vascular normalization augments necrosis and** 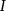 **cells.** (a)–(d) are simulations of the model, including AT + PD, without the Jain vascular normalization; AA therapy, initiated at the arrow, reduces angiogenesis. (e)–(h) are simulations of the model, including AT + PD, such that angiogenesis is enhanced from the start of AA therapy (red arrow) for a transient period of time (red arrow to black arrow). (a) and (e) plot the total mass of 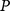 cells. (b) and (f) plot the percent necrosis (*ie* areas including>90% necrosis). (c) and (g) plot the total mass of 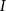 cells. (d) and (h) plot percent brain invasion (*ie* brain including>10^−4^ I cells). Units are arbitrary.(TIF)
