# Supplementary material for: Effects of Anti-Angiogenesis on Glioblastoma Growth and Migration: Model to Clinical Predictions
Source: PLoS One. 2014 Dec 15;9(12):e115018. doi: 10.1371/journal.pone.0115018 (PMC4266618; doi:10.1371/journal.pone.0115018)
Supplement: S1 Text — Legend of clinical imaging of Figs. 2d–2s . (PDF) [file pone.0115018.s001.pdf]

Information S1

Elizabeth Scribner[1] Olivier Saut[2] Paula Province[3] Asim Bag[4]

Thierry Colin[2] Hassan M Fathallah-Shaykh[1,3]

[1]Department of Mathematics, The University of Alabama at Birmingham, Birmingham, Alabama, United States of America, [2] Department of Mathematics, University of Bordeaux, Talence, France, [3]Department of Neurology, The University of Alabama at Birmingham, Birmingham, Alabama, United States of America, [4]Department of Radiology, The University of Alabama at Birmingham, Birmingham, Alabama, United States of America.

Legend of clinical imaging of Figures 2d-2s

Patient 10 of Table 1 (Left panel, d through k): Primary GBM on this female patient was diagnosed at the age of 44. The postcontrast T1 weighted image (d) and FLAIR image (h) at the time of first recurrence, approximately 6 months after the initial resection of the tumor, demonstrate large nodular enhancing tumor in the left superior frontal gyrus (arrow) with significantly large peritumoral FLAIR abnormality (arrow). The postcontrast T1 weighted image (e) and FLAIR image (i) obtained 2.5 months after the start of the bevacizumab therapy demonstrate almost complete resorption of the enhancement (arrow) and significantly improved FLAIR abnormality (arrow). The postcontrast T1 weighted image (f) and FLAIR image (j) obtained 4.5 months after start of the bevacizumab therapy demonstrate expansion of the necrosis without any worsening of enhancement (arrow) associated with interval worsening of FLAIR abnormality (arrow). The postcontrast T1 weighted image (g) and FLAIR image (k) obtained at the time of recurrence (8 months after the start of bevacizumab therapy) demonstrates irregular thick rind-like enhancement at the margin of the tumor (arrow) with further worsening of the peritumoral FLAIR abnormality (arrow).

Patient 7 of Table 1 (Right panel, l through s): Primary GBM on this male patient was diagnosed at the age of 55. The postcontrast T1 weighted image (l) and FLAIR image (p) at the time of first recurrence approximately 7 months after the initial resection of the tumor demonstrate nodular enhancing tumor around the left frontal horn (arrow) with significantly large peritumoral FLAIR abnormality (arrow). The postcontrast T1 weighted image (m) and FLAIR image (q) obtained 2.5 months after the start of the bevacizumab demonstrate almost complete resorption of the enhancement (arrow) and significantly improved FLAIR abnormality (arrow). The postcontrast T1 weighted image (n) and FLAIR image (r) obtained 4 months after start of the bevacizumab demonstrate expansion of the necrosis without any worsening of enhancement (arrow) associated with interval worsening of FLAIR abnormality and mass effect to the left frontal horn (arrow). The postcontrast T1 weighted image (o) and FLAIR image (s) obtained at the time of recurrence (6 months after the start of bevacizumab) demonstrates irregular thick rind-like enhancement at the margin of the tumor (arrow) with further worsening of the peritumoral FLAIR abnormality (arrow).
