# Supplementary figures and images for: Effects of Anti-Angiogenesis on Glioblastoma Growth and Migration: Model to Clinical Predictions
Source: PLoS One. 2014 Dec 15;9(12):e115018. doi: 10.1371/journal.pone.0115018 (PMC4266618; doi:10.1371/journal.pone.0115018)

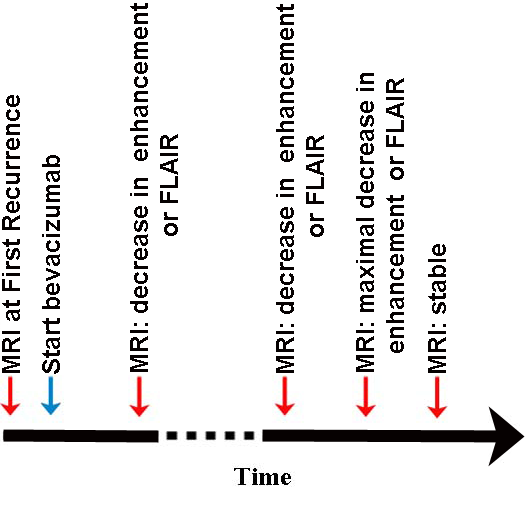

Supplement: S1 Figure — Selection of the 23/70 patients. Cartoon depicting the timing of the MRIs before and after first recurrence and the identification of 23/70 patients, treated by bevacizumab at first recurrence (see Table 1), who have a MRI with no new or increased enhancement after the MRI showing the maximal beneficial effects of bevacizumab on enhancement or FLAIR. (TIF) [file pone.0115018.s002.tif]

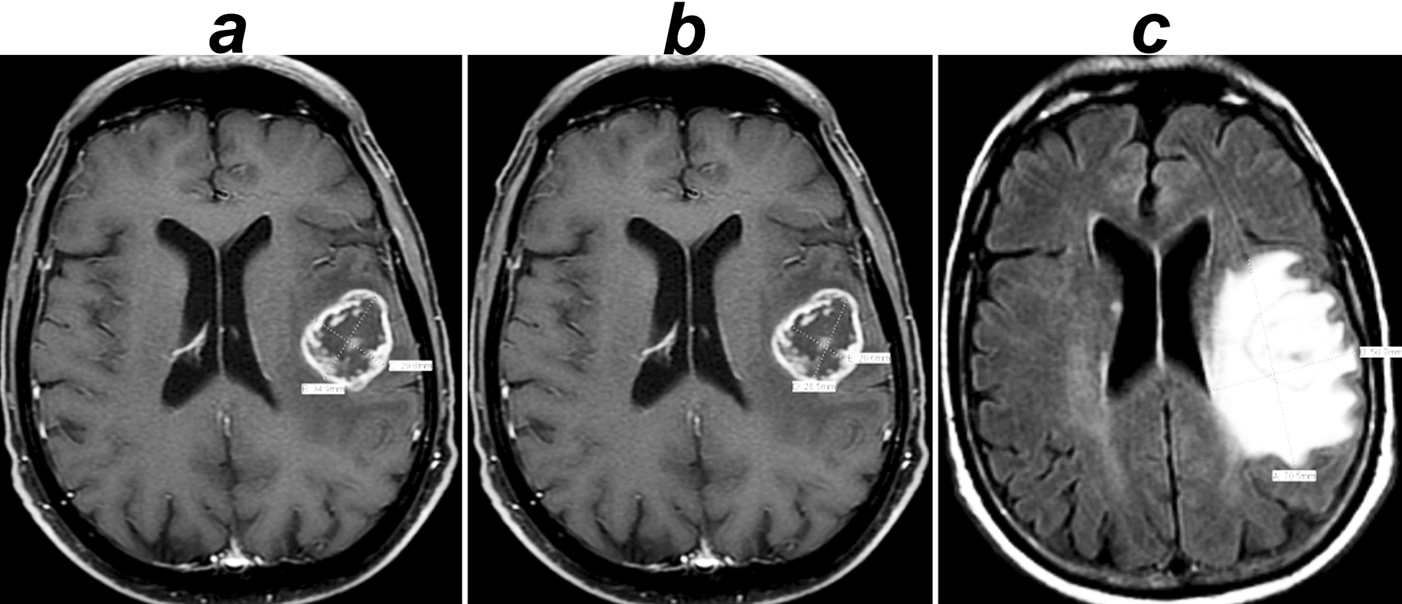

Supplement: S2 Figure — Tumor, Necrosis and FLAIR Measurements. An example of the measurements. (a) and (b) show the measurements of the largest diameters of the areas of enhancing tumor and necrosis, respectively. (c) shows the largest diameters of the areas showing FLAIR signal abnormality. The areas are computed by the product of the two perpendicular diameters. The measures plotted on the y-axis of Fig. 3i are FLAIR area - tumor area. (TIF) [file pone.0115018.s003.tif]

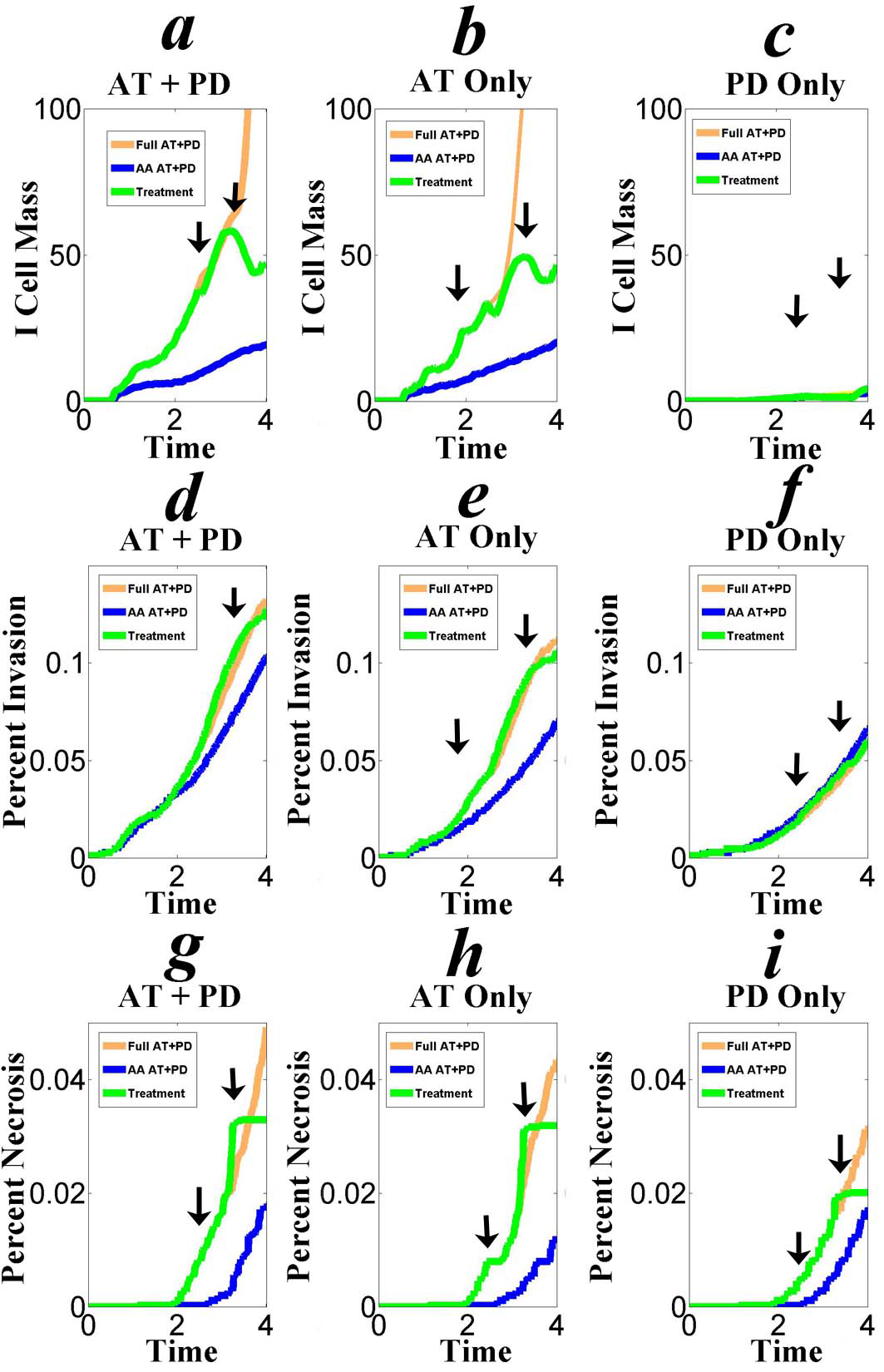

Supplement: S3 Figure — Comparison of the Treatment, Full, and AA Models. The Full, AA (ie starting from time 0), and treatment curves are colored in orange, blue, and green, respectively. The latter consists of the Full model until time step = 2500 (first black arrow) when AA is applied and then lifted at time step = 3500 (second black arrow). Simulations of the AT + PD model are shown in (a), (d), and (g). Simulations of the AT only model are shown in (b), (e), and (h). Simulations of the PD only model are shown in (c), (f), and (i). The effects on I Cell mass, Percent Brain Invasion (ie brain including>10−4 I cells), and Percent Necrosis (ie areas including>90% necrosis) are shown in (a–c), (d–f), and (g–i), respectively. The effects on P cell Mass is shown in Figs. 2(u)–2(w). Time units are arbitrary. (TIF) [file pone.0115018.s004.tif]

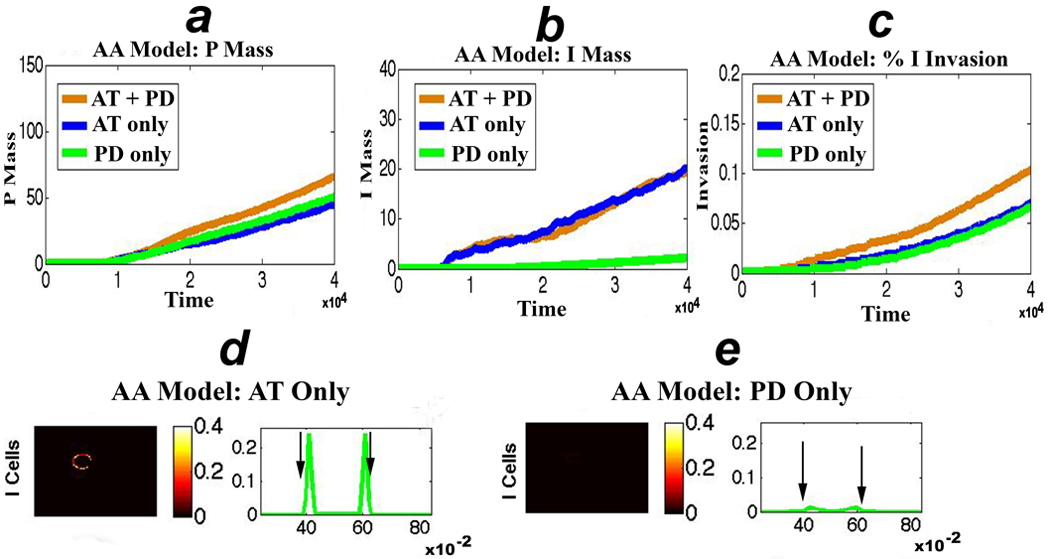

Supplement: S4 Figure — AT enhances brain invasion in the AA model. The first row compares the effects of AT only (blue), PD only (green), and AT + PD (orange) on the evolution of the proliferative tumor mass (a, P Mass), invasive tumor mass (b, I Mass), and percent brain invasion by I cells (c) for the AA model. (d) and (e) show the 2-dimensional distribution of cells at the final steps of the AT only and PD only models, respectively. The parameters parameters (AT) and (PD) are the same as in Figs. 3a–3h. Units are arbitrary. (TIF) [file pone.0115018.s005.tif]

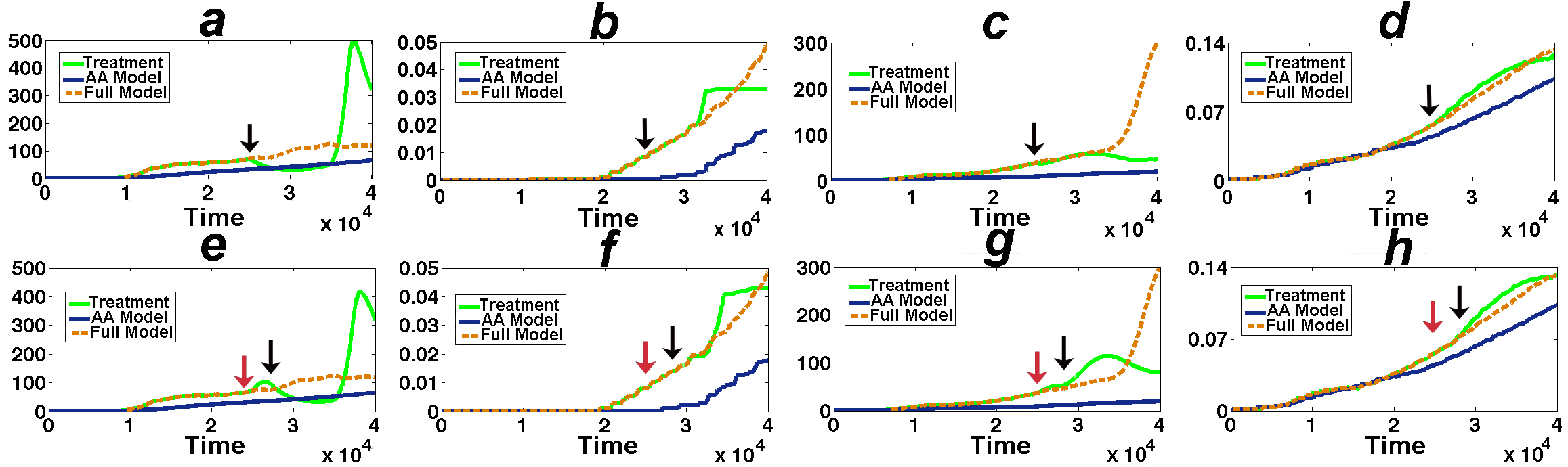

Supplement: S5 Figure — Jain vascular normalization augments necrosis and cells. (a)–(d) are simulations of the model, including AT + PD, without the Jain vascular normalization; AA therapy, initiated at the arrow, reduces angiogenesis. (e)–(h) are simulations of the model, including AT + PD, such that angiogenesis is enhanced from the start of AA therapy (red arrow) for a transient period of time (red arrow to black arrow). (a) and (e) plot the total mass of cells. (b) and (f) plot the percent necrosis (ie areas including>90% necrosis). (c) and (g) plot the total mass of cells. (d) and (h) plot percent brain invasion (ie brain including>10−4 I cells). Units are arbitrary. (TIF) [file pone.0115018.s006.tif]
